# Phage-induced diversification improves host evolvability

**DOI:** 10.1186/1471-2148-13-17

**Published:** 2013-01-22

**Authors:** Hywel TP Williams

**Affiliations:** 1 College of Life and Environmental Sciences, University of Exeter, Exeter, Devon, UK

## Abstract

**Background:**

Bacteriophage (viruses that infect bacteria) are of key importance in ecological processes at scales from biofilms to biogeochemical cycles. Close interaction can lead to antagonistic coevolution of phage and their hosts. Selection pressures imposed by phage are often frequency-dependent, such that rare phenotypes are favoured; this occurs when infection depends on some form of genetic matching. Also, resistance to phage often affects host fitness by pleiotropy (whereby mutations conferring resistance affect the function of other traits) and/or direct costs of resistance mechanisms.

**Results:**

Here a simple model of bacteria and bacteriophage coevolving in a resource-limited chemostat is used to study the effect of coevolving phage on the evolution of bacterial hosts. Density-dependent mortality from phage predation limits the density of any single bacterial strain, preventing competitive exclusion by faster-growing strains. Thus multiple strains can coexist by partitioning resources and stable high diversity is created by negative frequency-dependent selection from phage. Standing bacterial diversity promotes adaptation in dynamic environments, since it increases the likelihood of a pre-existing genotype being suited to altered conditions. In addition, frequency-dependent selection for resistance creates transient local trade-offs between growth rate and resistance that allow bacterial strains to adapt across fitness valleys. Thus bacterial populations that (in the absence of phage) would have been trapped at sub-optimal local peaks in the adaptive landscape are able (in the presence of phage) to reach alternate higher peaks than could have been reached by mutation alone.

**Conclusions:**

This study shows that reasonable assumptions for coevolution of bacteria and phage create conditions in which phage increase the evolutionary potential of their hosts. Thus phage, in contrast to their deleterious effects on individual host cells, can confer an evolutionary benefit to bacterial populations. These findings have implications for the role of phage in ecosystem processes, where they have mainly been considered as a mortality factor; these results suggest that on long timescales phage may actually increase bacterial productivity by aiding the evolution of faster-growing strains. Furthermore, these results suggest that the therapeutic use of phage to treat bacterial infections (phage therapy) could have unintended negative side-effects.

## Background

Bacteriophage (viruses that infect bacteria) are the most abundant replicating entities on Earth, involved in processes at scales from global biogeochemical cycles [1,2] to the human gut [3] to the control of bacterial infection in medical [4-6] and industrial [7] applications. Rapid evolution of phage and their hosts imply that evolutionary dynamics are likely to be a factor in many natural and applied scenarios; thus understanding how phage affect the evolution of host bacteria is of key importance. Close interaction between phage and their hosts often leads to significant antagonistic coevolution [8-10]. While it is hard to generalise, there is good empirical [11-15] and theoretical [16,17] evidence that in many cases coevolution between bacteria and bacteriophage leads to host diversification. This raises the question of how such diversification affects the overall evolutionary trajectory of host bacteria that are also evolving under other selection pressures, e.g., from environmental conditions and resource competition. When coevolving traits do not affect other functions, coevolution and evolution of functional traits may be orthogonal and proceed independently. However, coevolving traits often appear to have a significant impact on host growth and/or reproductive rate [11,12,14,18], so that coevolutionary and evolutionary processes interact.

**Figure 1:**
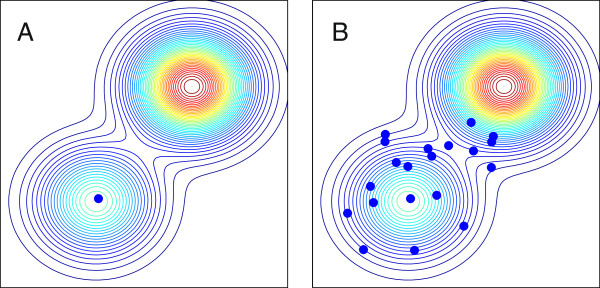
**How frequency-dependent selection from specialist phage might affect host adaptation when resistance and growth rate are pleiotropically linked.** Dots show the location of host strains on an underlying adaptive landscape shown by the contour lines. **A:** In the absence of phage, resource competition leads to a host population tightly converged on a suboptimal local peak in the adaptive landscape. Although a higher peak exists, hosts are unable to reach it due to an intervening fitness valley. **B:** Density-dependent phage predation creates frequency-dependent selection that causes hosts to diversify, so that the host community explores the adaptive landscape. Some strains cross the fitness valley and can now adapt to the higher peak.

Evolution is often visualised as movement of a population on an ‘adaptive landscape’ [19] which associates a fitness value with each genotype in some genetic space. A commonly discussed phenomenon is that populations can become converged on local peaks in the adaptive landscape and thus prevented from reaching higher peaks by intervening ‘fitness valleys’. Here it is proposed that the diversifying effect of specialist phage offers a mechanism by which host populations can adapt across fitness valleys to reach globally higher levels of fitness. This can be visualised by the thought experiment shown in Figure 1, which shows how phage-driven diversification might alter host adaptive dynamics. Diversification in response to phage action allows the host community to sample a larger region of the adaptive landscape than mutation alone, increasing the likelihood of discovering higher fitness peaks. The pre-conditions for this mechanism to operate are (i) diversifying selection from phage, and (ii) some form of genetic linkage between resistance and fitness.

Diversification of bacteria in response to phage predation has been hypothesised as a possible explanation for naturally observed high prokaryote diversity [13,15,20-24] and inferred from laboratory studies and genomic data [11,13,14]. Theoretical models of planktonic food-web ecology predict that selective viral predation can maintain host diversity by preventing dominance of host types that would otherwise monopolise available resources (the ‘kill-the-winner’ model [20,21]). The creation and maintenance of host diversity can be facilitated by high specificity of phage infection, whereby each phage strain is specialised on a limited range of hosts. This creates negative frequency-dependent selection that favours rare host genotypes. Theoretical models showing diversifying selection include matching-alleles models of infection genetics [16] and lock-and-key models of coevolutionary adaptive dynamics [17]. Empirical evidence for phage-induced diversification of bacteria comes from recent studies demonstrating fluctuating selection dynamics [25], in which there is continual reciprocal adaptation of both partners without directional change in overall infectivity or resistance [24,26]. Other laboratory coevolution studies have shown that phage can increase allopatric diversity in spatially structured host populations due to local adaptation [22,27,28].

Meanwhile, empirical data shows that resistance is often linked to host growth and/or reproductive rate [11,12,14,18,29], so that different resistance phenotypes have differing fitness in the absence of phage. There are two ways in which host traits involved in resistance might affect growth rate: (i) pleiotropy at related genetic loci, and (ii) costs associated with resistance mechanisms. If coevolving traits are subject to pleiotropy, then diversification of resistance traits will also create diversity in linked traits. Alternatively, if different resistance phenotypes have different growth costs, diversification will lead to selectable variation in replication rate. Either way, growth rate and resistance make distinct (though linked) contributions to overall fitness, and the diversifying effect of specialised phage might affect evolution of growth-related traits.

The scientific question addressed by this paper is how phage-host coevolution affects host evolutionary dynamics when specialist phage impose frequency-dependent selection. The coevolutionary study is based on an ecological model of bacteria and phage in a resource-limited chemostat, linked to an evolutionary model (inspired by the natural example of bacterial uptake receptors that are attachment sites for bacteriophage) in which host growth rate and resistance traits are pleiotropically linked [17,30]. This model is described in the next section. Results are then given showing that frequency-dependent selection from phage creates stable high diversity in hosts, whereby resources are partitioned between multiple subpopulations maintained by the balance between resource competition and predation. The standing diversity thus created allows the host community to respond quickly to environmental changes, improving adaptive capacity in dynamic environments. Furthermore, local trade-offs between resistance and growth allow host strains to adapt across fitness valleys to reach higher fitness peaks, thus improving adaptation on rugged adaptive landscapes. Finally, some implications of these findings are discussed in the broader context of ecological and evolutionary theory.

## Methods

The model used represents bacteria and bacteriophage coevolving in a resource-limited chemostat, where infection rate is based on genetic similarity. Host resistance and growth rate are pleiotropically linked, inspired by (e.g.) bacterial uptake receptors that are also phage attachment sites. Random mutations introduce new strains into the ecological dynamics. This model is studied for several different underlying relationships between growth rate and resistance phenotypes. Dynamics of the model are numerically resolved. In contrast to analytical approaches to adaptive dynamics [17,31-33], and in accordance with observed rapid evolution of bacteria and phage [34], ecological and evolutionary timescales are not explicitly separated. Additional details on methods are given in Appendix Appendix A: Supplementary Methods. All symbol definitions and parameter values are given in Table 1.

**Table 1:** Model parameters and variable definitions

**Symbol**	**Description**	**Value**	**Unit**
*R*	Resource concentration	Variable	*μgml*^−1^
*N*_*i*_	Density of host strain *i*	Variable	*cellsml*^−1^
*V*_*i*_	Density of virus strain *i*	Variable	*virionsml*^−1^
*N*_*init*_	Initial host density	4.6×10^4^	*cellsml*^−1^
*V*_*init*_	Initial virus density	8.1×10^5^	*virionsml*^−1^
*ω*	Chemostat dilution rate	0.0033	*min*^−1^
*R*_0_	Resource supply concentration	2.2	*μgml*^−1^
*ε*	Resource conversion rate	2.6×10^−6^	*μgcell*^−1^
*γ*	Maximum resource uptake rate	0.0123	*μgmin*^−1^
*K*	Half-saturation constant	4	*μgml*^−1^
*δ*_*i*_	Growth scaling for host *h*_*i*_	Range [*δ*_*min*_,*δ*_*max*_]	*scalar*
*δ*_*min*_	Min. growth scaling factor	0.8	*scalar*
*δ*_*max*_	Max. growth scaling factor	1.2	*scalar*
*ϕ*	Maximum adsorption rate	0.104×10^−8^	*ml*(*mincell*)^−1^
*θ*_*ij*_	Ads. scaling for *v*_*j*_ on *h*_*i*_	Range [0,*ϕ*]	*scalar*
*β*	Burst size	71	*virions*
*h*_*i*_	Genotype of bacteria *i*	Range [0,1]	*scalar*
*v*_*j*_	Genotype of phage *j*	Range [0,1]	*scalar*
${ĥ}_{i}$	Resistance phenotype of bacteria *i*	Range [0,1]	*scalar*
${\hat{v}}_{j}$	Infection phenotype of phage *j*	Range [0,1]	*scalar*
*h*_*init*_	Initial bacteria genotype	0.2	*scalar*
*v*_*init*_	Initial phage genotype	0.2	*scalar*
*s*	Specificity of phage	100	*scalar*
*M*_*B*_	Host mutation rate	10^−6^	*cell*^−1^
*M*_*V*_	Virus mutation rate	10^−6^	*virion*^−1^
*σ*_*B*_	Std. dev. of host mut. range	0.01	*scalar*
*σ*_*V*_	Std. dev. of virus mut. range	0.01	*scalar*
*μ*_*B*_	Bacterial mutation size	Random variable	*scalar*
*μ*_*V*_	Phage mutation size	Random variable	*scalar*
Δt	Integration timestep	10	*min*
*T*	Simulation duration	20×10^6^	*min*
*ρ*	Resolution of genotype diversity	0.01	*scalar*
*L*	Chemostat volume	1	*ml*

Variables can change during a simulation, derived values are deterministically calculated from other variables/parameters. Numerical values are parameters fixed for the duration of a simulation.

### Multi-strain chemostat model

The model represents the growth and interaction of a diverse community of bacteria and bacteriophage growing in a well-mixed single-resource chemostat. The model scheme is a variant of a reasonably well-studied type originally formulated for single-strain studies (e.g. [12,35]). Here a multi-strain formulation is used in which mutations can introduce new variants of bacteria and phage, while uncompetitive strains are eventually removed by chemostat dilution (cf. [17,30]). This creates a simple model in which bacteria and phage phenotypes can evolve by natural selection.

Ecological state dynamics for resource concentration *R* and the density of each host *N*_*i*_ and phage *V*_*j*_population in the chemostat are governed by the following equations:

(1) $$\frac{\text{dR}}{\text{dt}}=-\omega (R-R_{0})-\underset{i}{\sum }\varepsilon \gamma \frac{RN_{i}\delta_{i}}{R+K}$$

(2) $$\frac{dN_{i}}{\text{dt}}=-\omega N_{i}+\gamma \frac{RN_{i}\delta_{i}}{R+K}-\underset{j}{\sum }\theta_{\text{ij}}N_{i}V_{j}$$

(3) $$\frac{dV_{j}}{\text{dt}}=-\omega V_{j}+\underset{i}{\sum }\beta \theta_{\text{ij}}N_{i}V_{j}$$

Resource concentration is determined by supply concentration *R*_0_, chemostat flow rate *ω*, and by the total uptake of resource by all bacterial populations (determined by their growth rate scaled by a resource conversion constant *ε*). The density *N*_*i*_ of the *i*^*th*^ bacterial population is controlled by washout, growth, and mortality from phage (lysis). A Monod-form uptake-limited growth model is used whereby population growth is determined as a function of resource concentration, maximum uptake rate *γ*, half-saturation constant *K*, and a genetically encoded scaling factor *δ*_*i*_. The density *V*_*j*_ of the *j*^*th*^ phage population is determined by washout and production. Phage production is determined as the sum of production on all available hosts, assuming fixed burst size *β* and adsorption rate *θ*_*ij*_for phage *j* on host *i* (burst size is the number of new phage particles produced at each lysis event, adsorption is phage attachment to an uninfected cell). Only single infections are permitted and lysis is instantaneous, i.e., there is no latent period.

### Evolutionary process

The evolutionary model incorporates a stochastic mutation process into the deterministic ecological dynamics described above. Each distinct bacterial genotype *h*_*i*_and phage genotype *v*_*j*_in the current community is instantiated as a population. Bacteria and phage both evolve within a one-dimensional genetic space, i.e., each distinct genotype can be represented by a point on a line and adaptation occurs by movement along the line. Normally distributed mutations are applied to every new cell and phage particle (see Appendix Appendix A: Supplementary Methods). Since diversity is binned and standard deviations are small, most mutations have zero effect and offspring inherit the parental genotype.

Following mutation, a new population that instantiates the novel genotype is added to the system. If the density of any population falls below 1 *cellml*^−1^or 1 *virionml*^−1^(possible due to the continuous nature of the mathematical abstraction), that population is assumed to be lost and is removed from the system. Thus the ecological dynamics of the chemostat determine which genotypes invade or go extinct, based on phenotypic traits, without explicit separation of evolutionary and ecological timescales.

Bacterial resistance and growth rate traits, and phage infection traits, are allowed to evolve during each simulation. All other traits are universally fixed, although some are experimentally manipulated between simulations. Bacterial genotypes *h* are mapped to phenotypic traits for resistance ĥ and growth rate *δ*. Phage genotypes *v* map to an infection trait $\hat{v}$. See Appendix Appendix A: Supplementary Methods for details of the genotype-phenotype mapping.

Growth rate is derived from genotype using several different mappings to test model behaviour in different scenarios. All mappings create a growth rate landscape that determines the growth rate scaling factor *δ* as a function of genotype *h*. Manipulations include varying the number of peaks in the growth rate landscape and varying the relative height of different peaks. Localised ruggedness is introduced (when used) by adding a random noise signal and smoothing the result to differing degrees. Details of how landscapes are derived are described fully in Appendix Appendix A: Supplementary Methods and also where appropriate in the presentation of results.

### Infection model

The infection model assumes that the likelihood of infection of a bacterial host with genotype *h*_*i*_ by a phage with genotype *v*_*j*_following contact depends on their genetic similarity; infection rate is maximised when *h*_*i*_=*v*_*j*_ and decreases as genetic distance |*h*_*i*_−*v*_*j*_| increases. This model can be viewed as instantiating phenotypic coevolution corresponding to a ‘relaxed lock-and-key’ scheme in which infection can occur with some degree of genetic dissimilarity [17].

The rate of infection is calculated as a Gaussian function of the distance between the resistance ĥ and infection $\hat{v}$ traits. Thus the adsorption coefficient *θ*_*ij*_, which sets the adsorption rate for phage *v*_*j*_on bacterial host *h*_*i*_, is calculated as:

(4) $$\theta_{\text{ij}}=\phi e^{-s{\left({ĥ}_{i}-{\hat{v}}_{j}\right)}^{2}}$$

where *ϕ* is the maximum possible adsorption rate and *s* is a sensitivity parameter that controls the host specificity of phage. Tuning the value of *s* alters the rate of decline in adsorption rate as dissimilarity increases. Every successful adsorption event is assumed to result in infection and instantaneous cell lysis, releasing a burst of *β* new phage particles.

## Results

The primary results presented here are from numerical simulations of the model described above, supported by steady-state analysis (given in Appendix Appendix B: Steady-state analysis) where appropriate. This section first addresses the diversifying effect of phage on the bacterial population, which underpins subsequent examination of the evolvability benefits conferred on bacteria by coevolving phage, and their sensitivity to model parameters. Full sensitivity analysis of model parameters is given in Appendix Appendix C: Sensitivity analysis.

### Bacterial diversity from density-dependent phage predation

**Figure 2:**
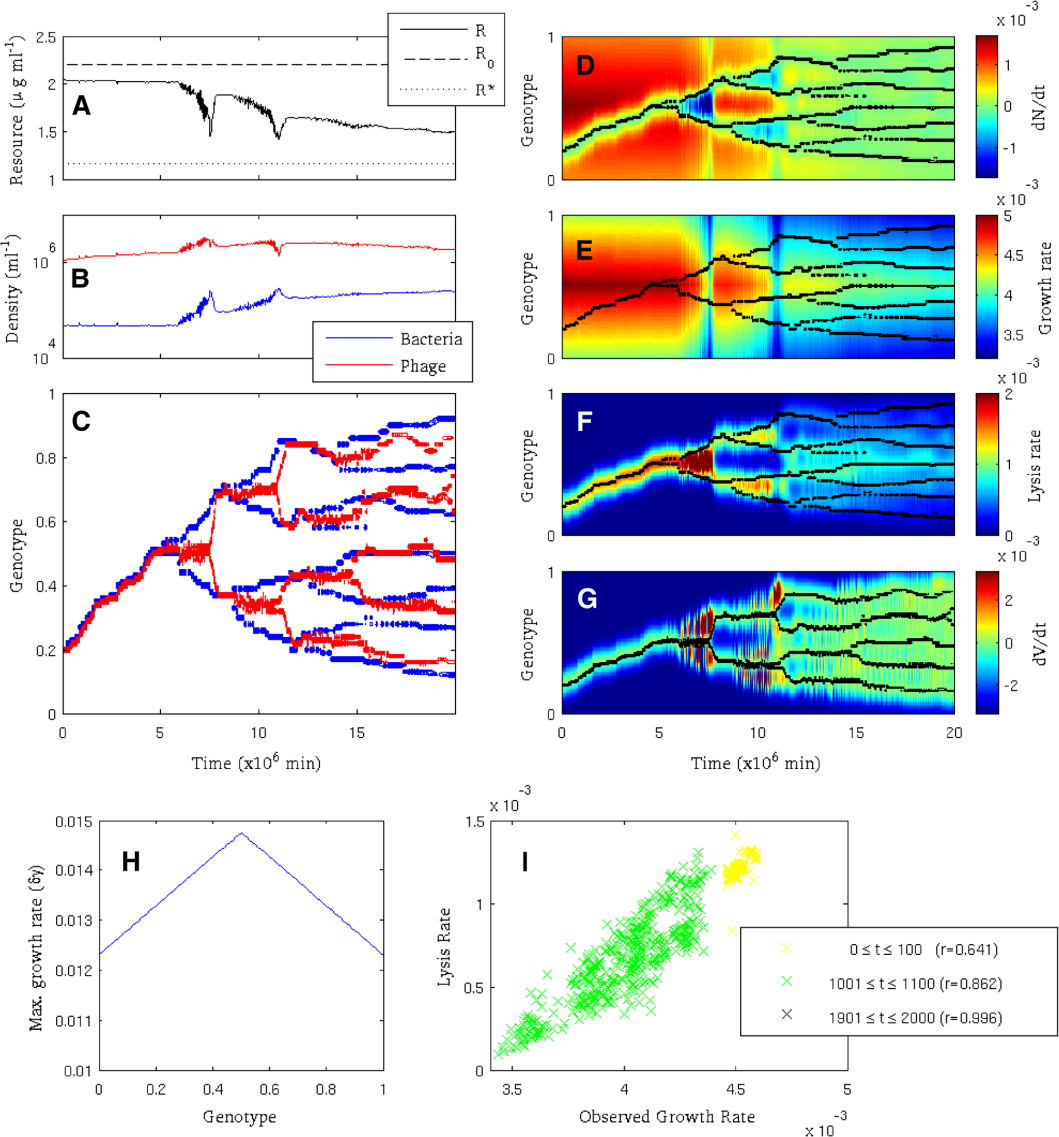
**Coevolution leads to diversification of bacteria and phage.** Plots show time series (A-C), adaptive landscapes with superimposed genotype density distributions (D-G), growth rate function (H), and growth-lysis correlations (I). **A**: Resource concentration *R*, supply *R*_0_, and *R*∗ for optimal growth phenotype. **B**: Total bacteria/phage density. **C**: Density distribution of bacteria and phage genotypes. **D**: Rate of change of bacterial genotype density ($\frac{\text{dN}}{\text{dt}}$). **E**: Bacterial genotype growth rate ($\delta \gamma \frac{R}{R+K}$). **F**: Bacterial genotype lysis rate ($\underset{j}{\sum }\theta_{j}V_{j}$). **G**: Rate of change of phage genotype density ($\frac{\text{dV}}{\text{dt}}$). **H**: Growth rate for bacterial genotypes. **I**: Correlation between growth and lysis rates for all bacterial strains forming >1*%*of total cell density, measured in three time intervals. All correlations (*r*) are significant with *p*<10^−19^in all cases. Parameter settings as Table 1.

To illustrate the negative frequency-dependent selection pressure imposed on bacteria by density-dependent phage predation, simulations were performed in which bacteria evolved on a smooth single-peak adaptive landscape. Figure 2 shows timeseries of resource concentration, total bacteria and phage density, and the density distribution of bacteria and phage in genotype space, together with fields showing bacteria/phage fitness landscapes over time, for an exemplar case study simulation. The simulation was initialised with a single bacterial host and perfect-match infectious phage (with genotype *h*_*init*_=*v*_*init*_=0.2) and run for a duration of *T*=20×10^6^*min*. Simulation parameters are given in Table 1.

The striking overall feature of this scenario is that there is rapid diversification of bacteria and correlated diversification of phage, corresponding to a pattern of frequency-dependent coevolution (Figure 2C). Consistent with ‘kill-the-winner’ ecological dynamics [20,21], specialised phage limit host density, preventing competitive exclusion and maintaining diversity, with resources partitioned between multiple strains. Phage predation selects for bacterial mutants with reduced susceptibility to infection by current dominant phage types. This creates diversifying selection on bacteria that leads to branching of the population into distinct clusters (hereafter strains) separated in genetic space by intervening regions in which mutants are susceptible to multiple phage strains and thus maladaptive. Host diversification selects for phage mutants that maintain infectivity by increasing genetic similarity to dominant bacterial strains. Phage therefore diversify to track their evolving hosts. Overall, bacterial populations act as attractors for phage in genetic space, while phage populations act as repellors for bacteria; the balance between these forces leads to the genetic dispersal of strains.

Diversification is ultimately limited by resource competition. As hosts diversify, the total host density increases substantially, with associated draw-down of resource concentrations (Figures 2A&2B). Without adaptation, the population density of a single bacterial host strain infected by perfect-match phage will tend to a lysis-limited steady state density that is significantly below the potential resource-limited carrying capacity of the system (Appendix Appendix B: Steady-state analysis). However, resource partitioning allows multiple bacterial strains to coexist and collectively draw down resource to limiting levels (Appendix Appendix B: Steady-state analysis). The value of *R*^∗^plotted in Figure 2A shows the resource concentration at which the fastest-growing (highest *δ*) bacterial strain would become resource-limited in the absence of phage (calculated using the method given in Appendix Appendix B: Steady-state analysis). Observed resource concentrations do not reach this theoretical minimum level, since the diverse community includes many strains with slower growth rates (and hence higher limiting concentrations) and most strains are phage-limited. However, total bacterial productivity is still ultimately limited by resource supply, when the slowest-growing (lowest *δ*) strain in the community (which does not reach sufficient density to support associated phage) reaches a resource-limited steady state. Strain diversity at steady-state depends on system parameters and is positively related to resource supply (Appendix Appendix B: Steady-state analysis).

Figures 2D and 2G show the fitness landscapes for bacteria and phage over time, found by calculating the growth rates of hypothetical genotypes in the current biotic and resource environment at each timepoint (see Appendix Appendix A: Supplementary Methods). Fitness landscapes for both bacteria and phage are highly dynamic, changing as a function of resource levels and the biotic environment. The overall selection pressure imposed on bacteria (visualised by gradients in the net rate of density change, $\frac{\text{dN}}{\text{dt}}$, Figure 2D) is determined by contributions from growth phenotype *δ*(selected via resource competition, Figure 2E) and resistance phenotype ĥ (selected via lysis, Figure 2F). The phage fitness landscape (visualised by gradients in the net rate of density change, $\frac{\text{dV}}{\text{dt}}$, Figure 2G) reflects the density distribution of bacteria, with positive growth only possible for phage genotypes with abundant hosts.

Within the evolutionary dynamics, three qualitatively different phases can be distinguished. Early in the simulation, bacteria can both increase growth rate and escape phage by adapting towards the fastest-growing phenotype (arbitrarily positioned at *h*=0.5, Figure 2H). This synergy drives rapid adaptation of bacteria, tracked by rapid adaptation of phage. When bacteria reach the optimal growth phenotype, they can only escape phage by adapting downhill in terms of growth rate (Figure 2E). This creates an evolutionary trade-off that allows diversification and coexistence of multiple strains. During this phase, repeated host divergence and phage counter-adaptation are observed, so that diversity of both phage and bacteria strains increases. Finally, as the system approaches steady state, there are no significant fitness gradients to drive adaptation of either bacteria or phage (i.e. $\frac{\text{dN}}{\text{dt}}\to 0$ and $\frac{\text{dV}}{\text{dt}}\to 0$ for all genotypes) and the genotype distributions are relatively constant over time (Figures 2D&2G).

The rate of bacterial adaptation (rate of change of genotype frequencies) is proportional to the deviation from a perfect linear correlation between growth and lysis across the bacterial community; the form of Equation 2 means that genotype density only changes when there is an imbalance between growth and lysis rates. This can be seen in Figure 2I, which shows the correlation between lysis and growth rates for all bacterial strains forming >1*%* of total density, observed during three time intervals at the beginning, middle, and end of the simulation. Imbalances can occur when mutation adds new bacteria/phage strains (e.g. if a novel bacterial strain arises with reduced susceptibility to current phage) but are reduced over time by ecological dynamics. Correlations increase over time, until at steady state, variation in growth rates is tightly correlated with variation in lysis rates, such that no net variation in fitness (net density change) is observed. In general, strong positive correlations are universally observed, showing that faster-growing host phenotypes experience greater levels of lysis; this highlights an ecological trade-off that allows multiple bacterial strains to coexist. Coexisting strains have varying growth rates, but any selective benefit from increased growth rate is balanced by a cost from increased lysis, resulting in kill-the-winner dynamics [20,21].

### Two evolvability benefits to hosts of specialised phage

**Figure 3:**
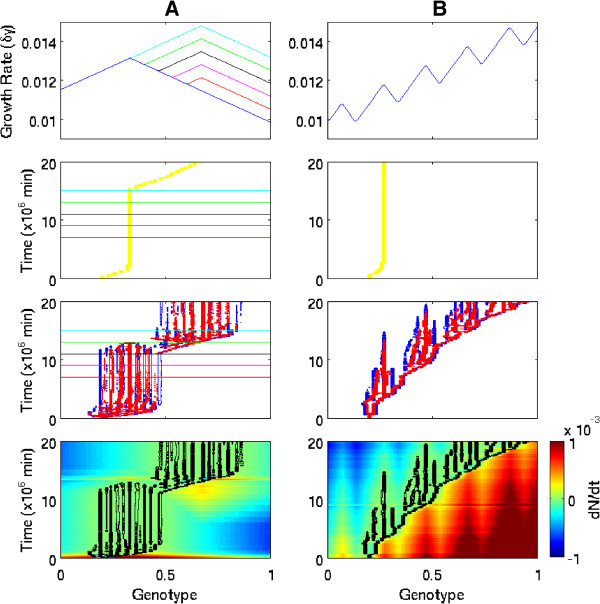
**Two evolvability benefits of coevolving phage.** Bacterial evolution on different adaptive landscapes with/without coevolving phage. **A:** Standing diversity aids adaptation in dynamic environments (colours show landscape change over time). **B:** Local trade-offs allow adaptation across fitness valleys on landscapes with multiple peaks. *Top:* Growth rate function, plotting maximum growth rate (*δγ*) as a function of genotype. *Middle:* Density contours showing distribution of bacterial and phage genotypes over time, for bacteria evolving alone (yellow), and for bacteria (blue) coevolving with phage (red). *Bottom:* Fitness landscape for bacteria coevolving with phage, plotting density contour (black) over field for rate of density change $\left(\frac{\text{dN}}{\text{dt}}\right)$. Parameter settings as Table 1 except for *M*_*V*_=10^−5^and *V*_*init*_=0 for no-phage treatments.

Having established the diversifying effect of specialist phage on host bacteria, the impact of diversification on bacterial evolution was explored. Figure 3 shows evolutionary dynamics for paired case study simulations of bacterial evolution with and without coevolving phage. Simulations are initialised with *h*_*init*_=*v*_*init*_=0.2 in each case; the only parameter difference is the initial density of phage (set to *V*_*init*_=0 for the no-phage case). Two forms of adaptive landscape were used to demonstrate two distinct evolvability benefits to hosts of diversification driven by coevolving phage. In the absence of phage, bacteria are selected by resource competition to maximise growth rate and thus increase fitness by local hillclimbing. Thus populations tend to become tightly converged on the nearest peak in the adaptive landscape. This leaves them unable to cross fitness valleys and on landscapes with multiple peaks they can become trapped on suboptimal local maxima. However, when forced to diversify by coevolving phage, it was found that: (i) standing diversity facilitates adaptation in dynamic environments, and (ii) local trade-offs between resistance and growth allow populations to adapt across fitness valleys.

#### Standing diversity facilitates adaptation in dynamic environments

The first scenario that was explored was bacteria evolving in a dynamic environment, using an adaptive landscape in which a second peak that sequentially increases in height was introduced alongside a static initial peak. Figure 3A visualises this dynamic landscape by colour-coding the landscape profile used at different timepoints (see Appendix Appendix A: Supplementary Methods). On the dynamic landscape, bacteria evolving alone and coevolving with phage quickly adapt to the initial peak. When evolving alone, the bacterial population is tightly converged on the initial peak, with a small amount of diversity provided by mutation-selection balance. When coevolving with phage, bacteria diversify to form a set of distinct strains distributed symmetrically around the peak.

The standing diversity produced and maintained by specialist phage can facilitate more rapid opportunistic adaptation to novel environments. As the second peak is introduced, a fitness valley is formed that separates the initial static peak from the new dynamic peak. As the relative height of the new peak is sequentially increased over time, eventually it reaches a stage where one of slowest-growing strains of the bacterial community is within mutation range of the lower slopes of the new peak. This strain then adapts rapidly towards the new peak, driven by synergistic selection pressures for increasing growth rate and reducing phage predation. A new diverse community then forms around the second peak, ultimately displacing the community around the initial peak due to resource competition. In contrast, the bacterial population evolving alone is unable to access the second peak until the fitness valley is completely removed, remaining trapped at the initial peak even when it becomes sub-optimal.

It is important to note that this is a benefit of diversity *per se* and is not unique to diversity created by phage; alternative mechanisms that preserve diversity might achieve a similar benefit. However, one particular advantage of phage-produced diversity is that it is maintained at steady-state by kill-the-winner ecological dynamics, whereas some other diversity-producing mechanisms (e.g., environmental change) might offer only transient diversity increases.

#### Local trade-offs between resistance and growth allow populations to adapt across fitness valleys

The next scenario was bacterial evolution on a rugged adaptive landscape, with multiple local peaks separated by fitness valleys on a slope of globally increasing growth rate (Figure 3B). As in the single-peak landscape example shown in Figure 2, the fitness benefit of escaping phage can counterbalance the fitness cost of moving downhill in terms of growth rate, allowing valleys to be traversed. On the multiple-peak landscape, bacteria diversify to form a fitness-neutral distribution in which most strains experience negligible selective gradients and do not adapt significantly once they arise. The only significant positive gradients are observed at the leading (uphill) edges of the strain distribution and this is where most adaptation (in the sense of a coherent subpopulation shifting its genetic composition) is observed, with the initial strain adapting steadily towards higher growth rates while new strains sequentially branch off. As the bacteria community climbs the landscape, slow-growing strains are lost at the trailing edge due to resource competition. As growth rates increase, more strains can enter the population (since higher growth rates enable growth at progressively lower resource concentrations) and bacterial strain diversity rises.

The proximate mechanism by which fitness valleys are crossed in this case study is a trade-off between local resistance and growth rate that allows host strains to adapt downhill in terms of growth rate. Effectively, the host population is pursued across the valley by coevolving phage; at each stage, the transient benefit of escaping phage outweighs the cost of reduced growth rate. The population always follows positive local gradients in the net rate of density change. While crossing a valley, this implies decreasing growth rates, compensated by the decreased lysis rates that result from reduced susceptibility to dominant phage. This mechanism is a dynamic (non-steady-state) effect of ecological trade-offs, and is distinct from the intrinsic benefit of steady-state diversity that is identified above.

### Controls on host diversification and evolvability

**Figure 4:**
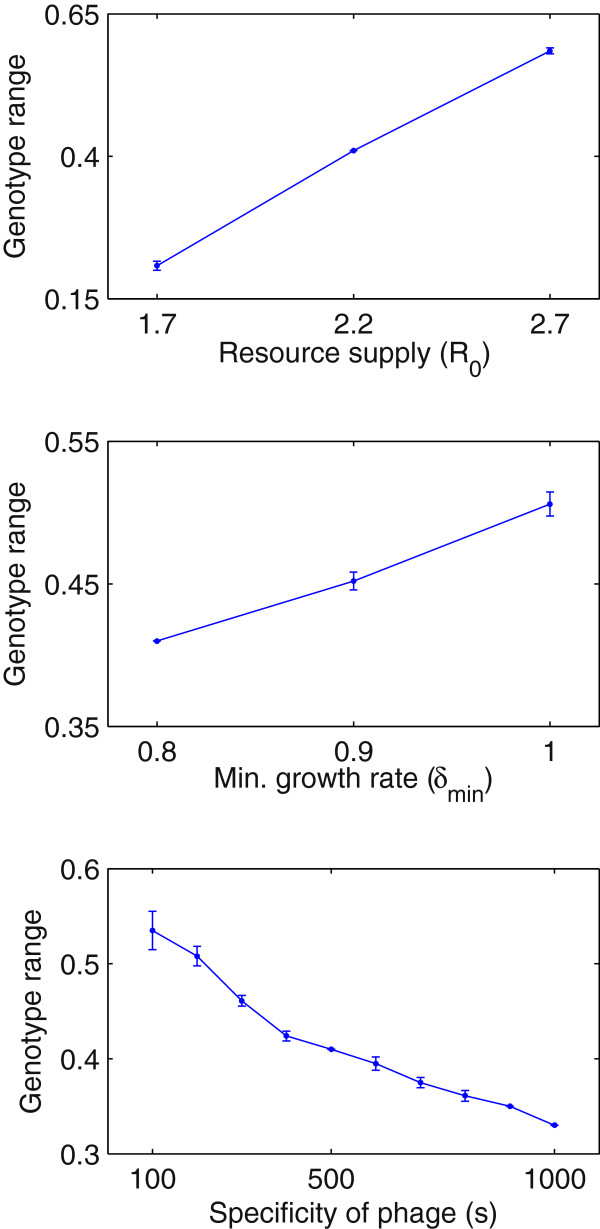
**Sensitivity of host genotype variation to resource supply*R***_***0***_**, minimum growth rate*δ***_***min***_**, and specificity of phage*s*.** Genotype range measured as difference between maximum and minimum values in final bacterial community from coevolutionary simulations on a single-peak landscape (e.g. Figure 2); *mean*±1*s.d.*plotted for 10-run ensembles for each parameter combination. Parameter settings as Table 1 except for manipulated variables *R*_0_∈{1.7,2.2,2.7}, *δ*_*min*_∈{0.8,0.9,1},*s*∈{100,200,*..*,1000} and *h*_*init*_=*v*_*init*_=0.5, *T*=5×10^6^*min*, *M*_*B*_=*M*_*V*_=10^−5^.

The ability to respond to a changed environment depends on the diversity of bacterial genotypes at steady state, which is determined by the balance between resource competition and phage predation; bacteria minimise dispersal away from the optimal growth phenotype, within the constraint of reducing phage infection (Appendix Appendix B: Steady-state analysis). Figure 4 shows the sensitivity of community-wide bacterial genotype variation with respect to resource supply (*R*_0_), the slope of the adaptive landscape (manipulated using *δ*_*min*_), and the host specificity (*s*) of phage. Variation was measured from coevolutionary simulations on smooth single-peak landscape (e.g. Figure 2H) using ensembles of simulation runs to account for stochastic effects. Simulations were initialised with a single bacterial genotype and matched phage genotype at the optimal growth phenotype (*h*_*init*_=*v*_*init*_=0.5) and run for *T*=5×10^6^*min*. Variation was measured as the total range of genotypes (*h*_*max*_−*h*_*min*_) in the final bacterial community for which the corresponding strain density represented >1*%* of the total community. The range of host genotypes at steady state is positively related to resource supply *R*_0_; more strains can be supported with greater available resource. Since the separation between strains is held roughly constant, adding more strains implies greater overall variation. Genotype range is negatively related to the gradient of the landscape. Thus for a fixed maximum growth rate *δ*_*max*_ it is positively related to the minimum growth rate *δ*_*min*_, which controls the cost of diversifying away from the optimal growth phenotype. Host genotype range is negatively related to the specificity *s* of the phage, i.e. negatively related to the rate of decline in infection rate with genetic dissimilarity.

**Figure 5:**
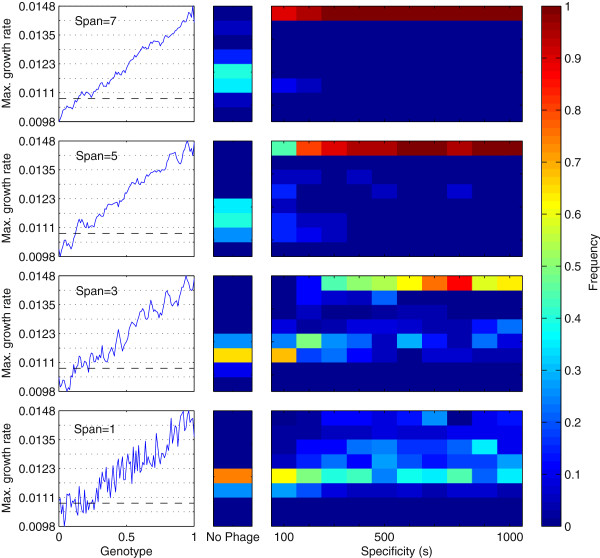
**Sensitivity of bacterial adaptation on rugged-slope landscapes to presence and specificity of coevolving phage.** Rows from top-to-bottom show increasing ruggedness (decreasing *span*). In each row, plots show: *Left:* Example of adaptive landscape, plotting maximum growth rate (*δγ*) as a function of genotype (blue solid line) and the growth rate of the initial bacterial genotype (black dashed line). *Middle:* Frequency distribution of highest maximum growth rates in final populations for bacteria evolving alone. *Right:* Frequency distribution of highest maximum growth rates in final populations for bacteria coevolving with phage, as a function of specificity of phage (*s*). See text for details of method. Ensemble sizes {35,33,20,20} for *span*={1,3,5,7} respectively. Parameter settings as Table 1 except for manipulated *s*, *M*_*B*_=*M*_*V*_=10^−5^and *V*_*init*_=0 for no-phage treatments.

To quantify the ability of phage to drive bacteria across fitness valleys using local trade-offs, ensembles of simulations were performed on rugged-slope landscapes formed by adding a uniform-random noise signal to a smooth linear slope (see examples in Figure 5), measuring sensitivity of bacterial adaptation to specificity of phage (*s*) and the ruggedness of the landscape (manipulated by varying the size *span* of the moving-average smoothing window used to create the landscape; see Appendix Appendix A: Supplementary Methods). For each value of *span*, an ensemble of landscapes was generated. On each landscape, a simulation was then performed with bacteria evolving alone, followed by multiple coevolutionary simulations varying the specificity parameter *s*. All simulations were initialised with the same seed strains (*h*_*init*_=*v*_*init*_=0.2) and run for *T*=20×10^6^*min*. Adaptation was measured by recording the maximum growth rate (highest *δ*) in the final bacterial community at the end of each simulation.

Figure 5 shows examples of the adaptive landscapes used and the frequency distribution of final maximum growth rates across each associated ensemble. For all levels of ruggedness (all values of *span*), bacteria evolving alone were able to achieve only a modest increase in maximum growth rate above the initial condition, typically becoming trapped at suboptimal local maxima. For almost all parameter combinations, the presence of coevolving phage offered a substantial improvement in adaptive performance, showing a significant shift towards a greater frequency of higher growth rates. For the smoother landscapes (higher *span*) the presence of phage often allowed the bacteria to reach the maximum possible growth rate. For the more rugged landscapes (lower *span*), the presence of phage increased the frequency of high growth rates, though instances still occurred when the population became trapped at suboptimal local peaks and performance was not significantly better than bacteria evolving alone. The most generalist phage (e.g. *s*={100,200}) offered little adaptive benefit on the most rugged landscapes, explained by the shallow gradient in lysis rates with increasing genetic distance relative to the steep local gradients in growth rate.

Overall, standing diversity (hence adaptive capacity in dynamic environments) is increased by generalist phage (that increase strain separation) and shallow growth rate gradients (that reduce the costs of diversifying away from the fastest-growing genotype). However, specialist phage offer the greatest evolvability benefit on rugged landscapes, since they can drive host populations across deeper fitness valleys.

## Discussion

The phage-driven establishment and maintenance at steady state of high bacterial diversity recapitulates kill-the-winner ecological dynamics [20,21], supporting arguments that such dynamics can arise by coevolution [13]. In the model used here, bacteria and phage coevolve until variation in lysis rates balances variation in growth rates, so that negative frequency-dependent selection gives way to emergent fitness neutrality at steady state. This can be seen in Figure 2, which shows that most bacteria/phage strains experience fitness gradients close to zero as steady state is approached, with tight correlations between growth and lysis rates. However, kill-the-winner dynamics are ongoing and do not require coevolutionary steady-state; Figure 2 also shows that there is always a strong correlation between growth and lysis, even during periods of rapid evolutionary change.

The high bacterial diversity maintained by phage at steady state in this model is consistent with explanations of observed hyper-variable regions in marine prokaryote genomes as the product of selection by phage predation [11,13,14,23,36]. The prediction of host diversification caused by phage is also supported by results from laboratory studies with coevolving bacteria and phage (e.g., [27,28]). However, in its current form (well-mixed, non-spatial) the model does not address the interesting further questions of how population structure, environmenal heterogeneity and dispersal might affect coevolutionary diversification [22,27,28].

Monotonic trade-offs between resistance and growth rate can occur in directional coevolution (e.g. when resistance is a generalised competence with a cost proportional to the range of phage strains resisted). In such scenarios, coevolving phage can only drive a decrease in host growth rates; as hosts evolve to become more resistant, their growth rate is reduced. Frequency-dependent coevolution does not permit monotonic trade-offs due to the specificity of infection and resistance; any increase in resistance to a given phage implies decreased resistance to other phage. However, here transient ecological trade-offs between growth rate and resistance relative to the current phage population were observed. Host mutants could temporarily reduce their susceptibility to phage predation, to an extent that permitted bacterial subpopulations to adapt downhill in terms of growth rate and cross valleys in the adaptive landscape. Furthermore, the local trade-offs that were observed allowed hosts to increase resistance by mutating in any direction that increased genetic distance, suggesting that selection for avoiding density-dependent phage predation should drive host exploration – and ultimately adaptation – on any underlying adaptive landscape.

Here specialised phage remove ruggedness in the local fitness landscape and allow coexistence of multiple fitness-neutral host strains. It is interesting to speculate that this ecologically mediated fitness neutrality might perform a similar role to neutrality in the genotype-phenotype map in molecular evolution, where the existence of multiple genotypes coding for the same phenotype improves evolvability by increasing the size of the single-mutation genetic neighbourhood and thus expanding the set of adjacent phenotypes [37,38]. Similar evolvability advantages might be gained in the coevolutionary scenario described here, where the fitness-neutral strain assemblages that result from frequency-dependent coevolution increase the set of adjacent genotypes by virtue of their genetic diversity.

The results presented here also have echoes of Sewall Wright’s hypothesised effect of “mass selection under changing conditions” [39], in which adaptation towards a novel adaptive peak in a changed environment creates a persistent genetic shift that orients the population towards an alternate peak when the original adaptive landscape is restored. Here the “changing condition” is the introduction of phage (or the introduction of novel phage), which degrade existing adaptive peaks due to the negative frequency-dependent selection they impose. If phage were removed from the coevolutionary scenario, the bacterial community would converge to the highest peak in the explored region of the adaptive landscape, consistent with Sewall Wright’s hypothesised mechanism. This theoretical prediction may be suitable for experimental testing; although previous attempts [40,41] did not observe a clear shift to a new fitness peak, the mechanistic reasons for this were not fully explored.

Biological evolution is far more complex than the simple model presented here, yet biological fitness landscapes are known to be highly dynamic and to often display multiple local optima, neutrality, and ruggedness. Here the presence of specialist phage performs a local smoothing of the fitness landscape, with differential predation increasing or reducing fitness from the baseline given by growth rate. This dynamic smoothing prevents the population from becoming trapped at local maxima in the adaptive landscape and thereby permits more effective adaptation towards global maxima. In addition, high standing diversity (maintained by frequency-dependent selection from phage predation) aids adaptation to changing environments. Thus over time the evolving bacterial community is able to discover progressively higher growth rates, which would be selectively favoured even in the absence of phage, but which could not have been discovered without the presence of coevolving phage. Thus coevolution with phage offers an ‘adaptive bridge’ to higher absolute growth rates that would not otherwise be reached.

## Conclusions

A simple model of coevolution between bacteria and bacteriophage was used to explore the impact of phage-driven diversification on bacterial evolution. Diversification of host resistance phenotypes significantly altered evolutionary dynamics of pleiotropically linked growth phenotypes, offering two distinct evolvability benefits: (i) the intrinsic benefit of higher diversity in adapting to novel environmental conditions, and (ii) a specific benefit from local trade-offs between resistance and growth that allowed adaptation across fitness valleys. Thus in scenarios where the key assumptions of the model are satisified (frequency-dependent selection from phage predation, genetic linkage of growth and resistance), phage can confer an evolutionary benefit on host populations. These findings have implications for the role of phage in ecosystem processes, since they predict that over long timescales the presence of phage would enable host bacteria to discover fitter genotypes with higher growth rates, with subsequent impacts on productivity, nutrient dynamics and biogeochemical cycles. The results also suggest that the use of phage to control bacterial infections in therapeutic or industrial applications might have unintended negative consequences by promoting more rapid bacterial adaptation.

## Appendix A: Supplementary Methods

### Mutation

At each integration timestep, the number of mutations is calculated based on the instantaneous production rate of new particles and the mutation rate (*M*_*B*_and *M*_*V*_for bacteria and phage respectively), scaled by chemostat volume *L*. Thus for bacterial genotype *h*_*i*_, the number of new mutants at each integration timestep is found as $M_{B}L\frac{\mathrm{\gamma R}N_{i}\delta_{i}}{R+K}$, while for viral genotype *v*_*j*_ the number of new mutants is $M_{V}L\underset{i}{\sum }\beta \theta_{\text{ij}}N_{i}V_{j}$. For each new cell or virion produced, the offspring genotype is found by adding a normal deviate to the parental genotype, that is, *h*_*mut*_=*h*_*i*_ + *μ*_*h*_(or *v*_*mut*_=*v*_*i*_ + *μ*_*v*_) where *μ*_*h*_(*μ*_*v*_) is a random value drawn from a normal distribution with mean 0 and standard deviation *σ*_*B*_(*σ*_*V*_).

### Genotype-phenotype mapping

Bacterial genotypes *h* are mapped to phenotypic traits for resistance ĥ and growth rate *δ*. Phage genotypes *v* map to an infection trait $\hat{v}$. Thus h→{ĥ,δ} and $v\to \hat{v}$. The mappings for resistance/infection traits are simple: ĥ=h and $\hat{v}=v$. In the absence of sufficient empirical data to generalise the relationship between host resistance and growth rate, this relationship is experimentally manipulated to explore associated coevolutionary dynamics. In all cases, a landscape is created that associates a growth rate with every bacterial genotype; this is done by mapping genotype *h*_*i*_∈[0,1] to growth rate scaling factor *δ*_*i*_∈[*δ*_*max*_,*δ*_*min*_], that is, *δ*=*f*(*h*) for some landscape function *f*.

Four kinds of adaptive landscape were used: smooth single-peak, dynamic two-peak, multiple-peak, and rugged-slope. The smooth single-peak landscape (see Figure 2) is a piecewise linear function of *h*, created by drawing straight lines on the (*h*,*δ*) axes to join point (0,*δ*_*min*_) to (0.5,*δ*_*max*_) to (1,*δ*_*min*_). The dynamic two-peak landscape (see Figure 3A) is found by taking the maximum value from two single-peak landscapes found as before; the second landscape becomes sequentially higher over time. The multiple-peak landscape (see Figure 3B) is created by composing the landscape of *n* repeated blocks. In each block, the mapping follows an up-down-up motif, i.e. within each block, straight lines join the points (0,0) to $\left(\frac{b_{w}}{3},b_{h}\right)$ to $\left(\frac{2b_{w}}{3},0\right)$ to (*b*_*w*_,*b*_*h*_), where $b_{w}=\frac{1}{n}$ is the block width and $b_{h}=\frac{\delta_{\text{max}}-\delta_{\text{min}}}{n}$ is the block height. The last point in each block is first point in the next, creating a saw-toothed increasing gradient. The rugged-slope landscapes (see Figure 4) are created by adding a uniform random noise signal (amplitude 0.2) to a smooth linear gradient from (0,*δ*_*min*_) to (1,*δ*_*max*_), then smoothing the result using a moving-average window of width *span*.

### Numerical method

Model code, integration and visualisation are performed in MATLAB; code is available on request. Simulations are initialised with a single bacterial population and infectious phage, then integrated forward for *T* minutes using a 4th order Runge-Kutta method with timestep Δt. Genotype diversity for both bacteria and phage is binned at a resolution of *ρ*, setting the minimum distinguishable genetic variation. The working range of possible genotypes for both bacteria and phage is set to be *h*,*v*∈[0,1]; simulation parameters are chosen such that genotype distributions stay within this range and simulations in which range edges are reached by mutation are discarded to avoid artefactual results.

### Calculating fitness landscapes

Figures 2 and 3 include plots of the fitness landscapes on which bacteria and phage evolve during the case study simulations. These are found by calculating the hypothetical net population growth rate for all possible genotypes at each timestep, to generate a field showing the expected rate of change of density for any genotype in the current environment over time. Assuming that the rate of change of genotype density is a measure for fitness, these fields correspond to time-dependent fitness landscapes for bacteria and phage.

The net rate of genotype density change for hypothetical bacterial genotype *h*_*x*_ at time *t* = *t*^′^ are calculated as a function of cell growth rate, lysis and washout for the current resource concentration and virus community, i.e. using Equation 2 to evaluate ${\left(\frac{dN_{x}}{\text{dt}}\right|}_{t=t^{\prime }}$ with values *R*(*t*^′^) and *V*_*j*_(*t*^′^). Growth rates are calculated as $\frac{\delta_{x}\mathrm{\gamma R}(t^{\prime })}{R(t^{\prime })+K}$ and expected lysis rates are calculated as $\underset{j}{\sum }\theta_{\text{xj}}V_{j}(t^{\prime })$. Similarly the net rate of genotype density change for hypothetical phage genotype *v*_*x*_ is predicted as a function of production and washout based on the current bacterial community composition, i.e. using Equation 3 to evaluate ${\left(\frac{dV_{x}}{\text{dt}}\right|}_{t=t^{\prime }}$ with values *N*_*i*_(*t*^′^).

The overlayed density contour shows the distribution of the bacteria and phage communities in the landscape, showing how they adapt over time in response to the dynamic fitness landscapes.

## Appendix B: Steady-state analysis

### Steady state density for a single bacteria strain without phage

For a single strain of bacteria in the absence of phage, Equations 1 and 2 simplify to:

(5) $$\frac{\text{dR}}{\text{dt}}=-\omega (R-R_{0})-\varepsilon \gamma \frac{\mathrm{RN\delta}}{R+K}$$

(6) $$\frac{\text{dN}}{\text{dt}}=-\mathrm{\omega N}+\gamma \frac{\mathrm{RN\delta}}{R+K}$$

Setting $\frac{\text{dN}}{\text{dt}}=0$ yields:

(7) $$\bar{R}=\frac{\mathrm{\omega K}}{\gamma \delta -\omega }$$

as the steady-state value for *R*. Setting $\frac{\text{dR}}{\text{dt}}=0$ yields:

(8) $$\bar{N}=\frac{-\omega \left(\frac{\mathrm{\omega K}}{\gamma \delta -\omega }-R_{0}\right)\left(\frac{\mathrm{\omega K}}{\gamma \delta -\omega }+K\right)}{\varepsilon \gamma \delta \left(\frac{\mathrm{\omega K}}{\gamma \delta -\omega }\right)}$$

as the steady-state value for *N*. For *δ*=*δ*_*max*_, this gives the lowest possible value of $\bar{R}$ (here defined as *R*^∗^) and the highest possible value for $\bar{N}$ (here defined as *N*^∗^). *N*^∗^ is the resource-limited carrying capacity of the system and *R*^∗^is the limiting resource concentration when it is reached.

### Steady state density of a single bacterial strain with phage

For a single strain of bacteria with associated perfect-match phage, Equations 2 and 3 simplify to:

(9) $$\frac{\text{dN}}{\text{dt}}=-\mathrm{\omega N}+\gamma \frac{\mathrm{RN\delta}}{R+K}-\mathrm{\theta NV}$$

(10) $$\frac{\text{dV}}{\text{dt}}=-\mathrm{\omega V}+\mathrm{\beta \theta NV}$$

Solving $\frac{\text{dV}}{\text{dt}}=0$ for *N* gives:

(11) $$\hat{N}=\frac{\omega }{\beta \theta }$$

as the steady-state density of bacteria limited by phage predation. Substitution of parameter values from Table 1 shows that $\hat{N}<\bar{N}$, thus phage limit bacterial growth below the resource-limited carrying capacity for reasonable values of *R*_0_. Equation 9 can also be solved for $\frac{\text{dN}}{\text{dt}}=0$ to give:

(12) $$\hat{V}=-\frac{\omega }{\theta }+\frac{\gamma }{\theta }\frac{\mathrm{R\delta}}{R+K}$$

showing that phage density is correlated with host growth rate at steady state.

### Steady state density for multiple bacterial strains with phage

To examine steady state densities for the multi-strain model, the system was simplified so that infection depends on a perfect genetic match, i.e., Equation 4 was replaced with:

(13) $$\theta_{\text{ij}}=\left\{\begin{array}{cc} \phi & \;\;\;\;\;\text{if}\;ĥ=\hat{v}\\ 0 & \;\;\;\;\;\text{otherwise} \end{array}\right)$$

Then Equations 1, 2 and 3 become:

(14) $$\frac{\text{dR}}{\text{dt}}=-\omega (R-R_{0})-\underset{i}{\sum }\varepsilon \gamma \frac{RN_{i}\delta_{i}}{R+K}$$

(15) $$\frac{dN_{i}}{\text{dt}}=-\omega N_{i}+\gamma \frac{RN_{i}\delta_{i}}{R+K}-\phi N_{i}V_{i}$$

(16) $$\frac{dV_{j}}{\text{dt}}=-\omega V_{j}+\beta \phi N_{j}V_{j}$$

By Equation 11 we have:

(17) $$\hat{N_{j}}=\hat{N}=\frac{\omega }{\beta \phi }$$

Note that $\hat{N}$ is a constant and holds for any bacterial strain with an associated phage, i.e. all hosts with infectious phage will have an equal density at steady state. Solving Equation 15 for $\frac{dN_{i}}{\text{dt}}=0$ gives:

(18) $$V_{i}=-\frac{\omega }{\phi }+\frac{\gamma }{\phi }\frac{R\delta_{i}}{R+K}$$

Since *R* is fixed at equilibrium, this shows that host density is constant for all *δ*_*i*_, while phage density varies positively with host growth rate.

### Calculating diversity at steady state

From the analysis above, the limiting resource concentration for a single strain *h*_*i*_ is given as $\bar{R_{i}}=\frac{\mathrm{\omega K}}{\gamma \delta_{i}-\omega }$. If strain *h*_*i*_ is introduced to the system, it can establish a population if $R>\bar{R_{i}}$, and will then (in the absence of infectious phage) draw down resource to the limiting concentration ${\bar{R}}_{i}$. Thus strain *h*_*i*_ will competitively exclude any strain *h*_*j*_where $\bar{R_{i}}<\bar{R_{j}}$ (i.e. where *δ*_*i*_>*δ*_*j*_).

Now suppose there exists a set of *H* possible host strains that each have unique growth rate scaling factors, labelled *h*_1_,*..*,*h*_*H*_ such that *δ*_1_>*δ*_2_>*..*>*δ*_*H*_. Since $\bar{R_{i}}$ depends inversely on *δ*_*i*_, it follows that ${\bar{R}}_{1}<{\bar{R}}_{2}<\mathrm{..}<{\bar{R}}_{H}$. Thus in the absence of phage, strain *h*_1_should competitively exclude all other strains. However, in the presence of infectious phage, the density of strain *h*_*i*_ is limited to $\hat{N}$ and thus strain *h*_*i*_ is unable to draw down resource concentrations to ${\bar{R}}_{i}$. Instead, resource concentration will stabilise at some $R^{\prime }>\bar{R_{i}}$ and strain *h*_*i*_ will only competitively exclude other strains *h*_*j*_where $R^{\prime }<\bar{R_{j}}$.

For simplicity and without loss of generality, assume that (i) host strains are introduced into the system in order of decreasing growth rate, and (ii) novel strains quickly acquire an associated phage. Then strain *h*_1_ can enter the system if $R_{0}>{\bar{R}}_{1}$ and will grow to phage-limited density $N_{1}=\hat{N}$, drawing down resource to a phage-limited equilibrium concentration, here labelled ${\hat{R}}_{1}$. Then strain *h*_2_ can enter the system if ${\hat{R}}_{1}>{\bar{R}}_{2}$, drawing down resource further to concentration ${\hat{R}}_{2}$, and so on. To generalise, let ${\hat{R}}_{i}$ be the equilibrium resource concentration when all strains *h*_1_,*h*_2_,*..*,*h*_*i*_have all established in the system and reached their phage-limited equilibrium density, such that $N_{1}=N_{2}=\mathrm{..}=N_{i}=\hat{N}$. The value of ${\hat{R}}_{i}$ can be found by solving Equation 14 at equilibrium for the conditions $N_{1}=N_{2}=\mathrm{..}=N_{i}=\hat{N}=\frac{\omega }{\beta \phi }$ and *N*_*i* + 1_=*..*=*N*_*H*_=0. This gives a quadratic in ${\hat{R}}_{i}$ which has one positive root given by:

(19) $${\hat{R}}_{i}\;=\;\frac{\left(R_{0}\;-\;K\;-\;\frac{\varepsilon \gamma \overset{i}{\underset{j=1}{\sum }}\delta_{j}}{\beta \phi }\right)\;+\;\sqrt{{\left(R_{0}\;-\;K\;-\;\frac{\varepsilon \gamma \overset{i}{\underset{j=1}{\sum }}\delta_{j}}{\beta \phi }\right)}^{2}\;+\;4R_{0}K}}{2}$$

Now suppose strains are added sequentially until the system reaches steady state and no further strains can invade. Let *x* be the number of coexisting host strains at steady state. Then strain *h*_*x*_is the slowest-growing strain in the final host community and it follows that $N_{x}<\hat{N}$ (since if $N_{x}\geq \hat{N}$, strain *h*_*x*_would support an associated phage population and become phage-limited at $N_{x}=\hat{N}$, thereby creating a niche for a slower-growing strain *h*_*x* + 1_). It also follows that ${\hat{R}}_{x-1}>{\bar{R}}_{x}>{\hat{R}}_{x}$. The expressions for ${\hat{R}}_{i}$ and ${\bar{R}}_{i}$ can be used to find *x* by iteratively comparing ${\hat{R}}_{i}$ and ${\bar{R}}_{i}$ for *i*=1,2,*..* until the case is found where ${\bar{R}}_{i}>{\hat{R}}_{i}$, which corresponds to strain *h*_*i*_ establishing a resource-limited population with $N_{i}<\hat{N}$, i.e. *h*_*i*_=*h*_*x*_ and *x*=*i*.

Note that this method does not depend on the order in which species are originally introduced; eventually the system will converge to the equilibrium condition described here, though transient dynamics would vary. To confirm this observation, suppose that there are two strains *h*_*a*_ and *h*_*b*_ that coexist at equilibrium. To show that the order in which *h*_*a*_and *h*_*b*_ were introduced does not matter, we need to show that *h*_*a*_ can establish in the system when *h*_*b*_ is already present, and vice versa. Let ${\hat{R}}_{a+b}$ be the equilibrium resource concentration when both strains are present and limited by phage. Let ${\hat{R}}_{a}$ and ${\hat{R}}_{b}$ be the phage-limited equilibrium resource concentrations for the system with *h*_*a*_ alone and *h*_*b*_ alone respectively. By the form of Equation 19 we know that ${\hat{R}}_{a}>{\hat{R}}_{a+b}$ and ${\hat{R}}_{b}>{\hat{R}}_{a+b}$. We know that ${\bar{R}}_{a}<{\hat{R}}_{a+b}$, and also that ${\bar{R}}_{b}<{\hat{R}}_{a+b}$, since if this were not true then the species would not coexist at equilibrium. Then by transitivity we have that ${\bar{R}}_{a}<{\hat{R}}_{a+b}<{\hat{R}}_{b}$ and also ${\bar{R}}_{b}<{\hat{R}}_{a+b}<{\hat{R}}_{a}$, meaning that either strain can establish a population at the phage-limited equilibrium resource concentration imposed by the other. Thus the order of introduction does not matter.

**Figure 6:**
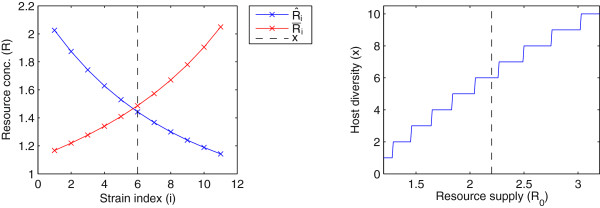
**Steady-state host strain diversity.** Plots show: *Left:* Graphical interpretation of the method for finding steady-state host strain diversity *x* as *x*=*i*where *i* is the lowest integer such that ${\bar{R}}_{i}>{\hat{R}}_{i}$. Dashed line shows the calculated bacterial diversity. *Right:* Steady-state host strain diversity *x* as a function of resource supply *R*_0_. Dashed line shows diversity for *R*_0_=2.2*μgml*^−1^. Both plots assume a pool of potential host strains *h*_1_,*..*,*h*_11_with unique growth scaling factors {*δ*_1_,*δ*_2_,*..*,*δ*_10_,*δ*_11_}={1.2,1.16,*..*,0.84,0.8}. Parameters as given in Table 1.

Figure 6A shows the sequential introduction method graphically, where *x* can be read as the minimum (integer) value of *i* for which ${\bar{R}}_{i}>{\hat{R}}_{i}$. Figure 6B shows steady state diversity found using this method for different values of *R*_0_ with an arbitrary set of candidate bacterial strains *h*_1_,*..*,*h*_11_with {*δ*_1_,*δ*_2_,*..*,*δ*_10_,*δ*_11_} = {*δ*_*max*_ = 1.2,1.16,*..*,0.84,*δ*_*min*_ = 0.8}, where *δ*_*min*_,*δ*_*max*_and other parameter values are as used for the simulations described in the main text (see Table 1). These results show that diversity is positively related to resource supply *R*_0_. The predicted diversity is 6 host strains for *R*_0_ = 2.2*μgml*^−1^as used for the main text, which is similar to the number of strains seen in Figure 2; however, the strict lock-and-key analysed here and the relaxed lock-and-key used in the main text simulations are not directly comparable.

### Stable coexistence with strict lock-and-key infection requires variation in host growth rates

As an interesting corollary, consider the case where all bacterial growth rates are equal, i.e., where *δ*_1_=*δ*_2_=*..*=*δ*_*H*_. Then ${\bar{R}}_{1}={\bar{R}}_{2}=\mathrm{..}={\bar{R}}_{H}=\bar{R}$. As before, a novel host strains can establish a population when $R>\bar{R}$. The method above can be used to calculate the maximum host diversity for the case in which all strains support phage. However, consider the first strain *h*_*x*_that enters the system and reaches a resource-limited steady state with $N_{x}<\hat{N}$, i.e. the first strain that becomes established but does not reach sufficient density to support phage. This strain will draw down resource to ${\bar{R}}_{x}=\bar{R}$, at which point $\frac{dN_{i}}{\text{dt}}<0$ for any strain with infectious phage, i.e. for all *i* < *x*, since all strains *h*_*i*<*x*_have associated phage populations which reduce their density. Thus their density will fall, so that $N_{i<x}<\hat{N}$ and $\frac{dV_{i}}{\text{dt}}<0$ for associated phage. Eventually either the host or the associated phage will go extinct, so that the only remaining bacterial strains are phage free. Thus the strict lock-and-key model implies that stable coexistence of hosts and phage cannot be achieved without variation in host growth rates.

## Appendix C: Sensitivity analysis

**Figure 7:**
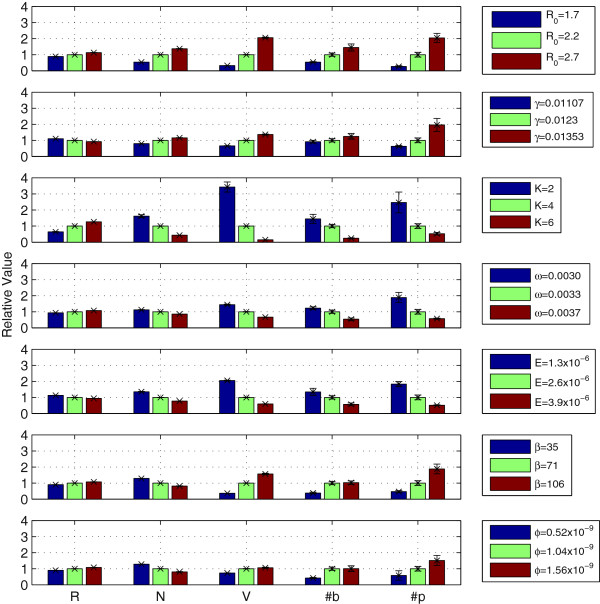
**Sensitivity of system state to model parameters.** Plots show sensitivity of system state variables to model parameters. Sensitivity shown as alteration of final state value normalised relative to benchmark parameters given in Table 1. Data shown are mean values (±1 std.dev.) from 10-run ensembles for each parameter set. State variables (left-to-right): resource concentration *R*, total bacterial density *N*, total phage density *V*, bacterial strain richness *#b*, phage strain richness *#p*. Model parameters (top-to-bottom): resource supply concentration *R*_0_, maximum resource uptake rate *γ*, half-saturation constant *K*, dilution rate *ω*, resource conversion rate *ε*, burst size *β*, maximum adsorption rate *ϕ*.

Here we present some sensitivity analysis on model parameters. Figure 7 shows how values of system state variables (resource concentration *R*, total bacterial density *N*, total phage density *V*, host strain diversity *#b*, phage strain diversity *#p*) are affected by increasing or decreasing various parameters (resource supply concentration *R*_0_, maximum resource uptake rate *γ*, half-saturation constant *K*, dilution rate *ω*, resource conversion rate *ε*, burst size *β*, maximum adsorption rate *ϕ*). These values were calculated as the ensemble mean across 10 runs with each parameterisation (to account for variation due to stochasticity in coevolutionary dynamics), taking the final value of each variable after a run of 20×10^6^ minutes. Results are shown relative to a benchmark parameterisation using the values given in Table 1. While some systematic quantitative effects of parameter variation are observed (Figure 7), no qualitative differences in the overall pattern of coevolution was observed. In all cases, coevolution lead to branching of both hosts and phage, with stable high diversity at steady state. Thus we conclude that the model results presented in the main text are robust to these parameter changes.

Another sensitivity test was performed on the mutation rates of bacteria *M*_*B*_ and phage *M*_*V*_. It was found (results not shown) that the pattern of coevolutionary branching and stable coexistence was conserved over wide ranges of values for *M*_*B*_ and *M*_*V*_. The only case when this pattern was disrupted was for *M*_*V*_ << *M*_*B*_(e.g. *M*_*V*_=10^−7^,*M*_*B*_=10^−5^); in this case the phage were unable to adapt fast enough to maintain infectivity on their evolving hosts. Eventually this resulted in phage extinction and reduction in host diversity by resource competition. The rate of bacterial mutation (*M*_*B*_) affected the time taken for the system to reach an evolutionary steady state, with lower values increasing the time to convergence.

A brief survey of mutation rates measured for bacteria and bacteriophage in the literature suggests that in natural systems, mutation rates for phage are typically orders of magnitude faster than those of their hosts [42,43]. Interestingly given the instability of coexistence in the current model when *M*_*V*_ << *M*_*B*_, experimental coevolution has shown that bacteria may increase their mutation rates in the presence of coevolving phage, with an effect of causing phage to go extinct [42]. However, for biologically plausible relative rates of host and phage mutation, we conclude that the results presented in the main text are robust.

## Competing interests

The author declares no competing interests.

## Author’s contributions

HW conceived this study, carried out model development and analysis, and wrote the manuscript.
